# White Light Emission from a Simple Mixture of Fluorescent Organic Compounds

**DOI:** 10.1038/s41598-019-47847-5

**Published:** 2019-08-14

**Authors:** Norfatirah Muhamad Sarih, Peter Myers, Anna Slater, Ben Slater, Zanariah Abdullah, Hairul Anuar Tajuddin, Simon Maher

**Affiliations:** 1 Department of Electrical Engineering and Electronics, University of Liverpool, Brownlow Hill, Liverpool L69 3GJ UK; 2 Department of Chemistry, Faculty of Science, University of Malaya, 50603 Kuala Lumpur, Malaysia; 3 Materials Innovation Factory, Department of Chemistry, University of Liverpool, Liverpool, L7 3NY UK

**Keywords:** Organic chemistry, Photochemistry, Green chemistry, Organic LEDs

## Abstract

Three fluorescent organic compounds—furocoumarin (FC), dansyl aniline (DA), and 7-hydroxycoumarin-3-carboxylic acid (CC)—are mixed to produce almost pure white light emission (WLE). This novel mixture is immobilised in silica aerogel and applied as a coating to a UV LED to demonstrate its applicability as a low-cost, organic coating for WLE via simultaneous emission. In ethanol solution and when immobilised in silica aerogel, the mixture exhibits a Commission Internationale d’Eclairage (CIE) chromaticity index of (0.27, 0.33). It was observed that a broadband and simultaneous emission involving coumarin carboxylic acid, furocoumarin and dansyl aniline played a vital role in obtaining a CIE index close to that of pure white light.

## Introduction

As display and lighting technology develops, efficient and environmentally friendly methods of producing coloured and white light are in increasing demand. Inorganic luminescence has long been established as a means to produce light with a range of colour profiles. However, most inorganic light-emitters are not environmentally friendly due to their high energy consumption and the process usually involving, liberating, or containing scarce metals or highly toxic chemicals like arsenic and cyanides. Organic fluorescent materials show excellent potential as a low-cost, green and sustainable alternative^1,2^. The most common application of organic fluorescence is for lighting purposes and there is much research surrounding other applications such as fluorescent labelling for bio-imaging^3–7^ and chemosensors^8–13^. Organic fluorescent materials have contributed significant advancements in the field of artificial lighting^14^. In particular, light emitting devices based on organic materials such as organic light-emitting diodes (OLEDs) and liquid crystal displays (LCDs) have received extensive attention in recent years^15,16^. Recent commercialisation of OLED technologies has further increased the demand for successful developments of organic white-light emitting systems^17,18^.

Several photo-physical principles have been used to achieve white light emission^14,19^, these include inter- and intramolecular charge transfer^20^, Forster resonance energy transfer (FRET)^2,21–23^, excited state intramolecular proton transfer (ESIPT)^24,25^, hydrogen bonding mediated J-aggregation^26^ and the mixing of monomer and excimer fluorescence^27^. Multi-component white light emission (WLE) comprises a mixture of different molecules that allow the colour temperature to be tuned by simply adjusting the composition, compared to using a single molecule^28^. Not surprisingly, there is significant recent interest for generation of mixed emitter WLE. Such systems comprise of inorganic, organic and hybrid systems including polymers^29^, metal-organic frameworks^30,31^, metal complexes^32^ and lanthanide doped systems^33^. Several examples of strategies employing mixtures of organic fluorophores have been reported, including a mixture of three emitting dyes covering the RGB region^34^, donor-acceptor conjugated pairs^22^ and self-assembly based compounds^35–39^. For instance, Wang *et al*. reported that micelle isolation can be used to inhibit FRET between fluorophores, thereby resulting in simultaneous emission of RGB dyes to produce WLE^40^. However, the WLE intensity is low due to the amount of fluorescent dyes encapsulated as the micellar core is restricted. Further to this, Wang and co-workers developed a new method to enhance the intensity of WLE by controlling FRET and micellar nanostructures such that the RGB intensities could be increased to produce simultaneous emission^41^. Others have mixed a range of oligomers that rely on intramolecular fluorescence energy transfer processes like FRET to produce tuneable WLE^38,42,43^. Our novel approach is to target a mixture of simple organic compounds that share a similar range of excitation wavelengths in the UVA region (340–375 nm) yet emit at different wavelengths in the visible range (400–700 nm). We hypothesise that a mixture of three such components should emit white light and this can be combined with a suitable, commercially available UV LED.

**Figure 1:**
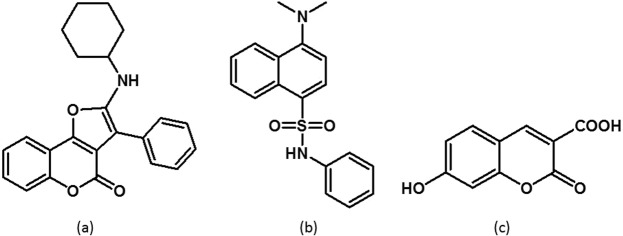
Chemical structures of (**a**) FC, (**b**) DA and (**c**) CC.

Three compounds were identified for this strategy: furocoumarin (FC), dansyl aniline (DA), and 7-hydroxycoumarin-3-carboxylic acid (CC) as shown in Fig. 1, respectively. These compounds yield distinct emission colours yet are all excited in the UVA region (340 to 375 nm). 7-hydroxycoumarin-3-carboxylic acid is commercially available and was used as purchased. The synthesis of furo[3,2-c]coumarin was reported by Nair and co-workers and involves a [4 + 1] cycloaddition with *in-situ* generated heterocyclic coumarin methides and isocyanides^44^. The synthesis of dansyl aniline was adapted from a procedure reported by Xiao *et al*. (see Materials and Methods)^45^.

## Results and Discussion

**Figure 2:**
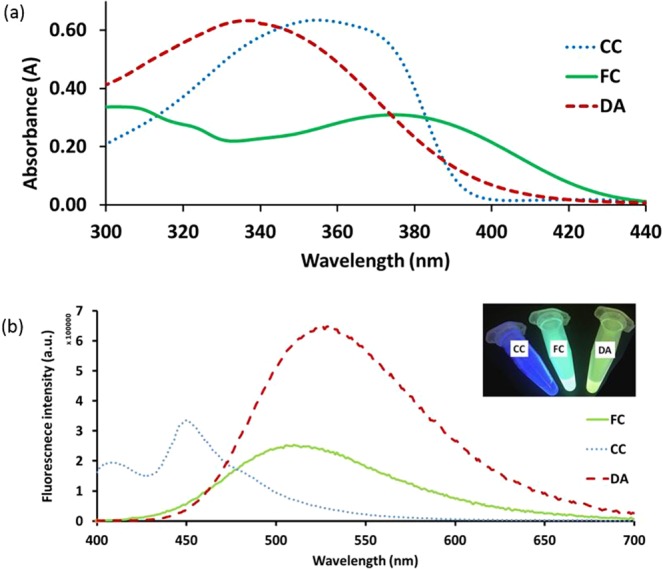
(**a**) Absorption spectra for each of the compounds (CC = 2.2 × 10^−5^ M, FC = 1.6 × 10^−5^ M and DA = 1.6 × 10^−5^ M) in ethanol. (**b**) Fluorescence spectra for each of the compounds (CC = 2.2 × 10^−3^ M, FC = 1.6 × 10^−3^ M and DA = 1.6 × 10^−3^ M) in ethanol. Inset: Photograph taken under UV light (390 nm) for each of the compounds (CC, FC and DA) in ethanol.

**Figure 3:**
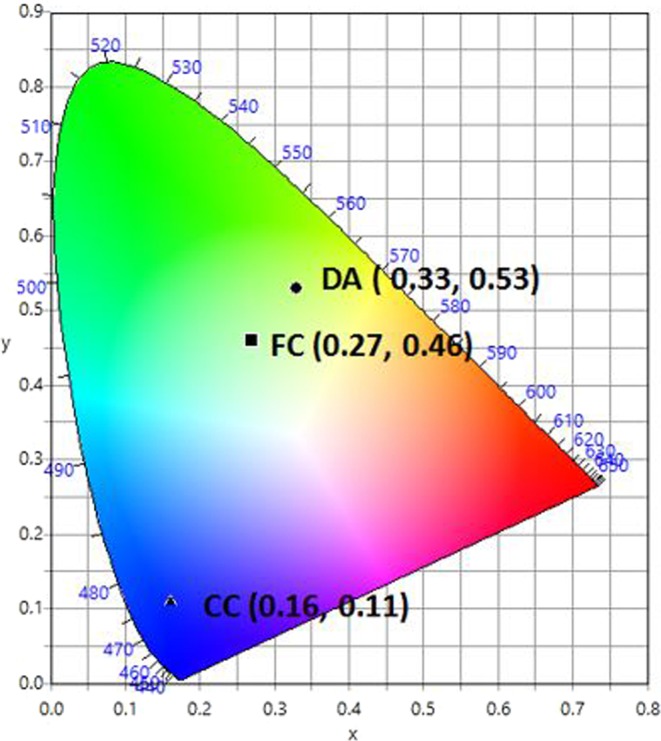
CIE-1931 diagram. Chromaticity plot for colour coordinates of CC (▲), FC (■) and DA (●).

Each of the three fluorescent organic compounds was prepared in requisite proportions (CC = 2.2 × 10^−3^ M, FC = 1.6 × 10^−3^ M and DA = 1.6 × 10^−3^ M) in ethanol and subjected to UV-Vis absorption and fluorescence spectral studies. UV-Vis absorption and fluorescence spectra for all of the fluorescents are shown in Fig. 2a,b, respectively. Each of the fluorescents has a broad characteristic absorption band of around 300–420 nm with varying maxima; 339 nm for DA, 355 nm for CC, and 375 nm for FC. Fluorescence emission maxima occur at 450 nm, 512 nm, and 530 nm for DA, CC, and FC, respectively. Under UV light excitation at 390 nm, CC, FC, and DA show blue, cyan and yellow emission, respectively (inset photograph, Fig. 2b). The CIE chromaticity coordinate for CC appears in the blue-violet region (0.16, 0.11); the coordinate for FC appears in the cyan region (0.27, 0.46); for DA it appears in the green-yellow region (0.33, 0.53) of the CIE diagram (Fig. 3). It is observed that the points for CC, FC and DA appear on the opposite side of the white region in the CIE diagram. From this observation it was hypothesised that it should be possible to obtain WLE by mixing the three fluorescent compounds.

**Figure 4:**
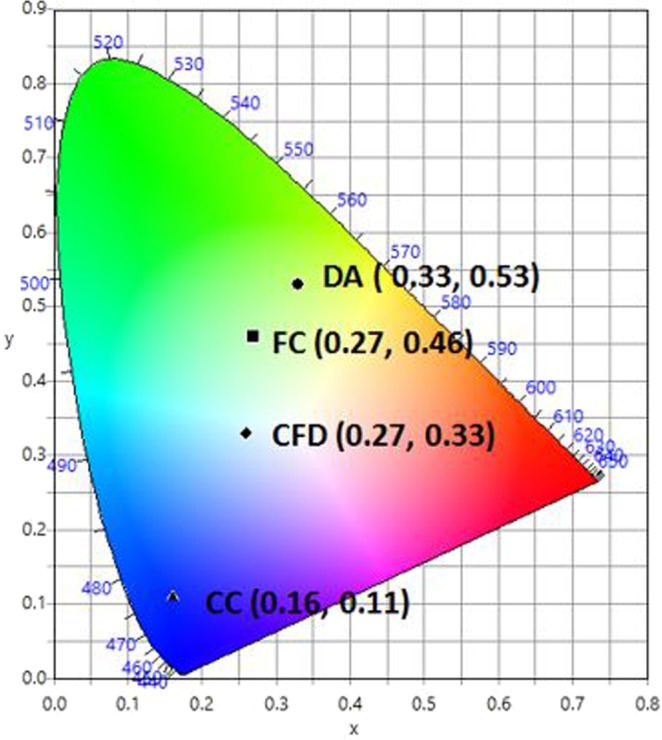
CIE-1931 diagram. Chromaticity plot for colour coordinates of CC (▲), FC (■), DA (●) and for the mixture, CFD (◆) corresponding to a ratio of 1.375:1:7 CC:FC:DA, in ethanol solution.

Accordingly, a series of solutions were prepared by varying the ratio of DA with a fixed ratio of CC and FC at 1.375:1 (Supplementary Table S1). Solutions were prepared containing varying ratios of CC:FC:DA (1.375:1:3, 1.375:1:4, 1.375:1:5, 1.375:1:6 and 1.375:1:7) and were analysed by UV-Vis absorption and fluorescence spectral studies as shown at Figs S1, S2a–c and S3. Based on the UV-Vis spectra, all the ratios show similar excitation wavelength, within the range of 350 to 355 nm. The mixture of the fluorescent solution was excited at various wavelengths (340, 350, 360, 370, 375, 380,390, 400 and 404 nm). Further increases in the concentration ratio up to 1.375:1:8 and 1.375:1:9 were also trialled but led to an increasingly yellow coloured emission profile, therefore no further fluorescence analysis was conducted at these increased ratios as it was deemed to be beyond the scope of the present research objective (to produce WLE). Each of the fluorescence spectra shows almost similar bands of emission and fluorescence intensity except at 390 nm, 400 nm and 404 nm. From these, 390 nm exhibits high fluorescence intensity with a broadband emission profile, producing almost pure white light emission. By varying the excitation wavelength it is expected that, for a mixture of different fluorophores, the emission profile should vary^46^. Figure 4 shows the colour coordinates for these solutions in the CIE diagram. It is seen that the point CFD (0.27, 0.33), corresponding to the composition concentration of CC = 2.2 × 10^−3^ M, FC = 1.6 × 10^−3^ M and DA = 1.6 × 10^−3^ M, with ratio of 1.375:1:7; CC:FC:DA, is extremely close to that of pure white light (0.33, 0.33).

**Figure 5:**
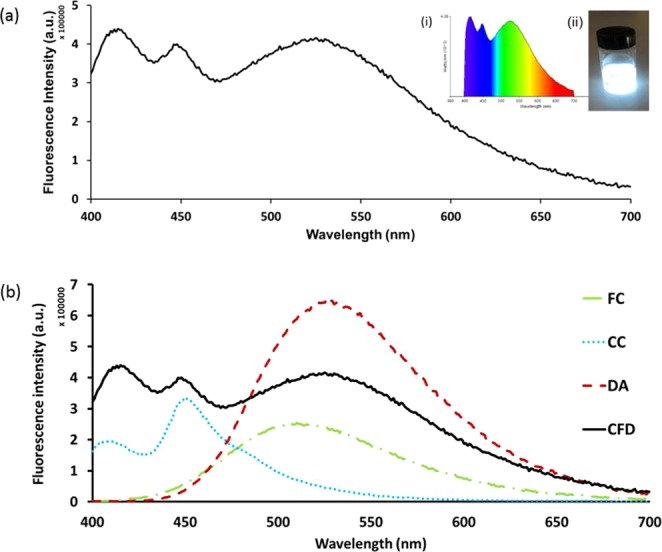
(**a**) Fluorescence spectrum for a mixture, CFD, of CC:FC:DA (1.375:1:7) in ethanol at 390 nm. Inset: (i) Colour spectrum and (ii) photograph of the CFD solution in ethanol under UV light (390 nm). (**b**) Shows overlay of both fluorescence spectrum from a mixture (CFD, where CC:FC:DA = 1.375:1:7) and the fluorescence spectra of the three components individually (FC, CC, DA) in ethanol at 390 nm.

The emission spectrum corresponding to point CFD covered the entire visible region (400–700 nm) (Fig. 5), with three emission maxima at 430, 450 and 525 nm. These bands appear to be similar to the individual CC, FC and DA emissions, indicating that the emission spectra are due to the simultaneous emission from all of the fluorescents. The fluorescence analysis from the mixture clearly shows that excitation at 390 nm results in emission that broadly covers the entire visible range up to about 700 nm. The absorption and emission spectra of the fluorescents do not exhibit any significant overlap (CC, FC and DA: absorption band (300–400 nm), emission band (400–700 nm)). Furthermore, by comparing the emission spectrum of the mixture CFD with the spectrum from each individual compound (Fig. 5(b)), there is very little/no peak shifting which indicates that simultaneous emission occurs which results in white light generation. Examining the individual emission responses of FC and CC (Fig. 5(b)) there is also the possibility that self-absorption may be occurring and it is expected that this may become more apparent at higher concentrations^47,48^. Observations from a complimentary experiment in which the amount of DA was increased 7-fold and added to a fixed CC:FC (1.375:1) ratio, given in (Table S1), show the production of WLE at 390 nm. Furthermore, the CIE coordinates, correlated colour temperature (CCT) and photograph under UV lamp illumination (390 nm) are given for various ratio combinations of the fluorescents in ethanol. As is seen in Table S1, simple variations in relative composition of the components modifies the CIE indices and the corresponding colour temperatures in a facile manner.

**Figure 6:**
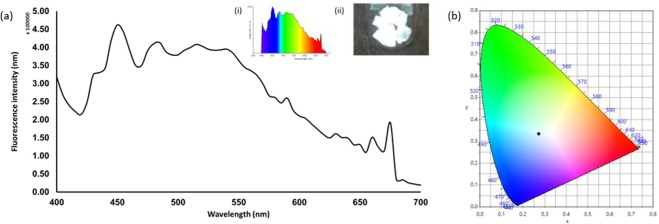
WLE aerogel. (**a**) Emission spectrum of mixture incorporated in to aerogel for WLE. Inset (i) Colour spectrum of the modified aerogel and (ii) photograph of the modified aerogel under UV illumination (390 nm). (**b**) CIE plot for colour coordinate of white light emitting aerogel (0.27,0.33).

**Figure 7:**
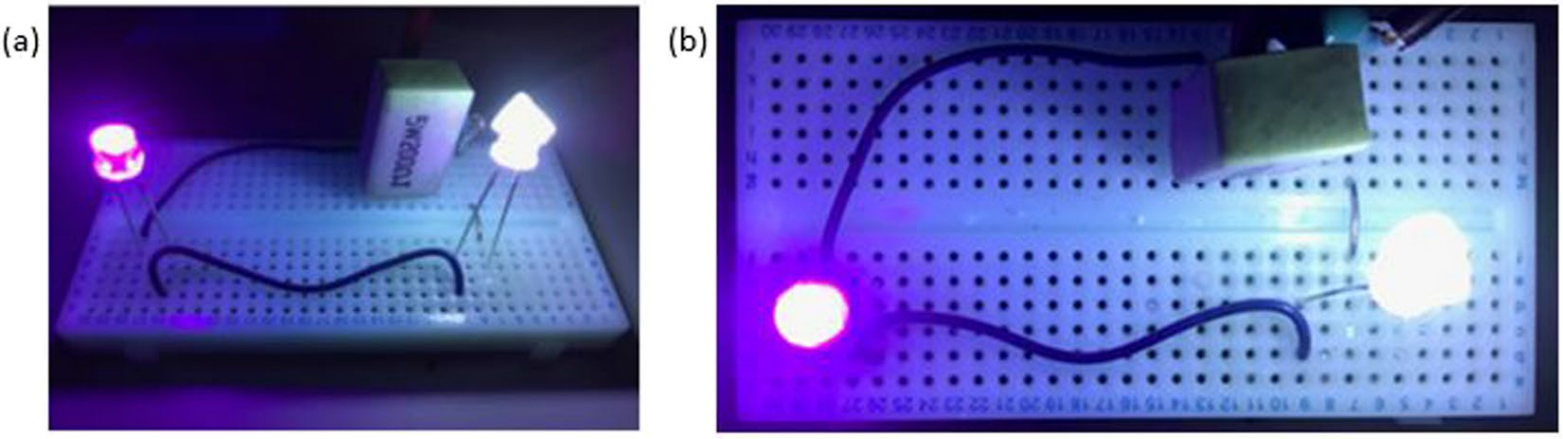
Side-by-side image for the same UV LED. In each image the left hand side is the uncoated UV LED and the right hand side LED is coated with aerogel to produce WLE. Both images are taken of the same experimental setup: (**a**) is a side view and (**b**) is a top view.

The possibility of producing WLE with this simple mixture from a solid media has also been explored. Aerogels offer high porosity (nanometer-scale pore sizes), they are lightweight materials with low densities (0.003–0.15 kg/m^3^) and large surface areas (500–1000 m^2^/g)^49,50^. Such properties mean that aerogel has great potential for use in a wide range of applications, including thermal insulation^51^, electrochemical applications (e.g., super capacitors)^52^, materials for tissue engineering^53^, bio-sensors^54^, amongst several others. In this work, we tested two commercially available aerogel variants: dry hydrophilic and dry hydrophobic silica aerogel. The hydrophilic aerogel produces white light upon excitation after soaking in the WLE mixture, whilst the hydrophobic aerogel produced a blue light emission. The aerogel was soaked in the WLE mixture in ethanol for 3 days in a fume hood. Subsequently, the gel was filtered and allowed to dry. The dried aerogel then exhibits WLE when excited under UV light illumination. The fluorescence emission spectrum of the mixture incorporated in to aerogel is shown in Fig. 6a, which covered the visible region from 400 to 650 nm. The aerogel shows good WLE under UV light (inset, Fig. 6a(ii)). A good CIE coordinate value of (0.27, 0.33) was obtained for the WLE aerogel from the corresponding emission spectrum, excited at 390 nm (Fig. 6b). Finally, as an exemplar application to demonstrate the potential of this approach, we applied the WLE aerogel in an ad-hoc fashion as a coating for a commercial UV LED. Side by side images of the regular UV LED and the modified aerogel WLE LED are shown in Fig. 7. As can be observed, the modified LED produces a uniform WLE.

## Conclusion

In summary, we have produced WLE from three simple organic compounds two of which were synthesised using a simple procedure. The optimised mixture of furocoumarin, dansyl aniline and 7-hydroxycoumarin-3-carboxylic acid at a ratio 1.375:1:7 in ethanol, generates almost pure white light, with identical CIE values in solution and when immobilised in solid silica aerogel, (0.27, 0.33). WLE from simple organic materials offers significant potential for applications in the global lighting industry. When immobilised in aerogel and applied to a commercial UV LED, it was demonstrated how this approach can produce effective WLE. Following this approach can open up further research avenues utilising aerogels to provide thermal insulation for solid state lighting applications. Furthermore, it would be interesting to see if such a system can be used as a dye for tuneable dye laser applications. To the best of our knowledge, this is a unique mixture of simple organic molecules that when combined can produce WLE in solution and also solid media (i.e., aerogel).

## Materials and Methods

All starting materials and reagents were purchased from Sigma Aldrich (UK). They were used without further purification. Hydrophilic aerogel was obtained from Cabot Corporation (USA) for WLE solid preparation. ^1^H NMR (400 MHz) spectra were measured on a Bruker Biospin DRX-600 spectrometer using TMS as an internal standard. 7-hydroxy-3-carboxylic acid coumarin (CC) (Mw = 206.15 g/mol, Sigma Aldrich) was used as a blue-purple emitting material. UV-vis absorption and fluorescence spectra in solution were recorded on a Cary 5000 UV-Vis-NIR Spectrophotometer from Agilent Technologies and FLS 1000 Spectrometer from Edinburgh Instruments, respectively. Excitation and emission monochromator band pass were kept at 1 nm using a quartz cell cuvette (1 × 1 cm). CIE colour coordinates have been calculated using freely available Osram Sylvania software.

### Synthesis of furo [3,2-c] coumarin (FC)

**Figure 8:**
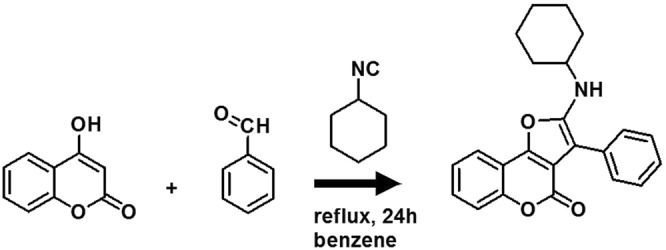
Synthesis of furo[3,2-c]coumarin derivatives.

4-hydroxycoumarin (4.86 g, 30 mmol, 1 eq.) and benzaldehyde (3.18 g, 30 mmol, 1 eq.) were dissolved in benzene (150 mL) and heated to reflux (Fig. 8). After 30 minutes, cyclohexyl isocyanide (3.27 g, 30 mmol, 1 eq.) was added to the reaction mixture, which was heated to reflux for a further 24 h. The pure compound was obtained by recrystallization from diethyl ether (100 mL) to yield a bright yellow crystalline powder (9.70 g, 90% yield). Analysis was in agreement with the literature^44^. Figure 8 shows the schematic of the reaction. Melting point = 110–112 °C, FTIR = 3289 (NH), 2930–2857 (cyclohexane), 1707 (C=O of pyrone), 1593 (C=C of pyrone). ^1^H NMR (400 MHz, CDCl3): δ = 7.80 (d, J = 7.27 Hz, 1H, Ar), 7.50 (d, J = 7.27 Hz, 2H, Ar), 7.43–7.45 (m, 4H, Ar), 7.34–7.35 (m, 2H, Ar), 4.23 (s, 1H), 3.60 (s, 1H), 1.26–2.01 (m, 10H). ^13^C NMR: δ 24.19, 25.54, 34.15, 53.69, 77.05, 97.47, 110.94, 112.87, 117.29, 119.49, 123.95, 124.89, 127.01, 128.66, 129.19, 130.73, 132.87, 149.81, 151.32, 154.89, 157.95. UV-Vis = 374 nm in ethanol. MS (ESI) = *m*/*z* 360.

### Synthesis of dansyl aniline (DA)

**Figure 9:**
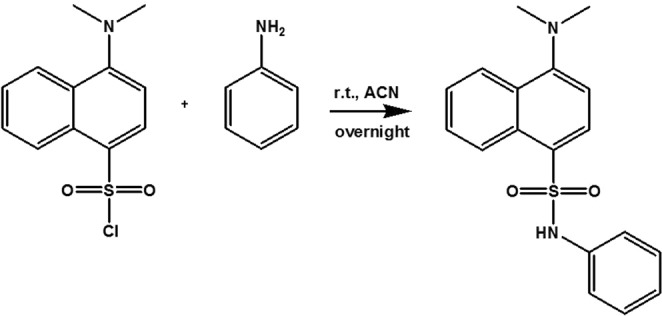
Synthesis of dansyl aniline.

Dansyl chloride (2.69 g, 10 mmol, 1 eq.) and aniline (9.30 g, 10 mmol, 1 eq.) were dissolved and stirred in acetonitrile (50 mL) overnight (Fig. 9). The pure compound was obtained by recrystallization from distilled water and ethanol to produce up to 65% yield (2.12 g). Analysis was in agreement with the literature^45^. Figure 9 shows the schematic of the reaction. Yellow powder, m.p. = 131–133 °C, FTIR = 3270 (NH), 2833 (N-CH_3_), 1601 (NH-aromatic), 1349 (SO_2_-NH), 1159 (SO_2_ stretching). ^1^H NMR (400 MHz, CDCl_3_): δ = 8.52 (d, 1H, J = 8.4 Hz, Ar), 8.35 (d, 1H, J = 8.8 Hz, Ar), 8.16 (d, 1H, J = 8 Hz, Ar), 7.61 (t, 1H, J = 7.6 Hz, Ar), 7.45 (t, 1H, J = 7.2 Hz, Ar), 7.22–7.20 (d, 1H, J = 8 Hz, Ar), 7.17–7.13 (m, 2H, Ar), 7.07–7.05 (m, 1H, Ar), 6.95–6.93 (d, 2H, J = 8 Hz, Ar), 6.68 (s, 1H), 2.90 ppm (s, 6H, N(CH_3_)_2_). ^13^C NMR: δ162.96, 152.18, 138.79, 133.17, 132.29, 132.11, 130.83, 130.39, 129.16, 128.61, 125.40, 121.80, 117.88, 115.22, 77.33, 77.02, 76.70, 45.41. UV-Vis = 339 nm in ethanol. MS (ESI) = *m/z 327*.

## Supplementary information

Supplementary Information
