# Supplementary material for: White Light Emission from a Simple Mixture of Fluorescent Organic Compounds
Source: Sci Rep. 2019 Aug 14;9:11834. doi: 10.1038/s41598-019-47847-5 (PMC6694174; doi:10.1038/s41598-019-47847-5)
Supplement: Supplementary file 1 — Supplementary Information [file 41598_2019_47847_MOESM1_ESM.docx]

**Supporting Information**

**White Light Emission from a Simple Mixture of Fluorescent Organic Compounds**

Norfatirah Muhamad Sarih^1,2^, Peter Myers^3^, Anna Slater^3^, Ben Slater^3^, Zanariah Abdullah^2^, Hairul Anuar Tajuddin^2^ and Simon Maher^1^*

^1^Department of Electrical Engineering and Electronics, University of Liverpool, Brownlow Hill, Liverpool, L69 GJ, UK.

^2^Department of Chemistry, Faculty of Science, University of Malaya, 50603 Kuala Lumpur, Malaysia.

^3^Materials Innovation Factory, Department of Chemistry, University of Liverpool, Liverpool, L7 3NY, UK.

*For correspondence, email: [s.maher@liverpool.ac.uk](mailto:s.maher@liverpool.ac.uk)

**Table S1**. Various ratio combinations of dansyl aniline (DA) with furocoumarin (FC) and carboxylic coumarin (CC)

| Series No. | CC  (2.2x10^-3^M) | FC  (1.6x10^-3^ M) | DA  (1.6x10^-3^M) | Excitation wavelength  (nm) | CIE  (x,y) | CCT (K) | Photograph under UV lamp (390 nm) |
| --- | --- | --- | --- | --- | --- | --- | --- |
| A | 1.375 | 1 | 3 | 340 | (0.21,0.25) | 34558 | 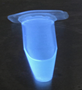 |
|  |  |  |  | 350 | (0.21,0.25) | 34558 |  |
|  |  |  |  | 375 | (0.21,0.24) | 49898 |  |
|  |  |  |  | 380 | (0.22,0.26) | 25581 |  |
|  |  |  |  | 390 | (0.23,0.27) | 22043 |  |
| B | 1.375 | 1 | 4 | 340 | (0.21,0.26) | 28475 | 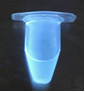 |
|  |  |  |  | 350 | (0.21,0.25) | 34558 |  |
|  |  |  |  | 375 | (0.21,0.25) | 34558 |  |
|  |  |  |  | 380 | (0.22,0.28) | 19499 |  |
|  |  |  |  | 390 | (0.24,0.29) | 14638 |  |
| C | 1.375 | 1 | 5 | 340 | (0.22,0.28) | 19499 | 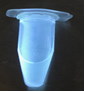 |
|  |  |  |  | 350 | (0.22,0.27) | 22043 |  |
|  |  |  |  | 375 | (0.22,0.27) | 22043 |  |
|  |  |  |  | 380 | (0.23,0.27) | 19890 |  |
|  |  |  |  | 390 | (0.24,0.30) | 14638 |  |
| D | 1.375 | 1 | 6 | 340 | (0.23,0.30) | 14638 | 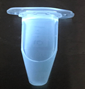 |
|  |  |  |  | 350 | (0.23,0.30) | 14638 |  |
|  |  |  |  | 375 | (0.23,0.28) | 13610 |  |
|  |  |  |  | 380 | (0.23,0.28) | 13610 |  |
|  |  |  |  | 390 | (0.25,0.31) | 11315 |  |
| E | 1.375 | 1 | 7 | 350 | (0.24,0.30) | 13610 | 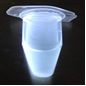 |
|  |  |  |  | 375 | (0.24,0.29) | 14683 |  |
|  |  |  |  | 380 | (0.24,0.33) | 11474 |  |
|  |  |  |  | 390 | (0.27,0.33) | 9167 |  |

**Figure S1**. Absorbance spectra for all the ratios tested in ethanol (A-E). A-E entries correspond with the description given in Table S1.

(a)

(b)

(c)

**Figure S2.** Fluorescence spectra for three different ratio mixtures: (a) 1.375:1:5 (series C, Table S1), (b) 1.375:1:6 (Series D, Table S1) and (c) 1.375:1:7 (Series E, Table S1).

**
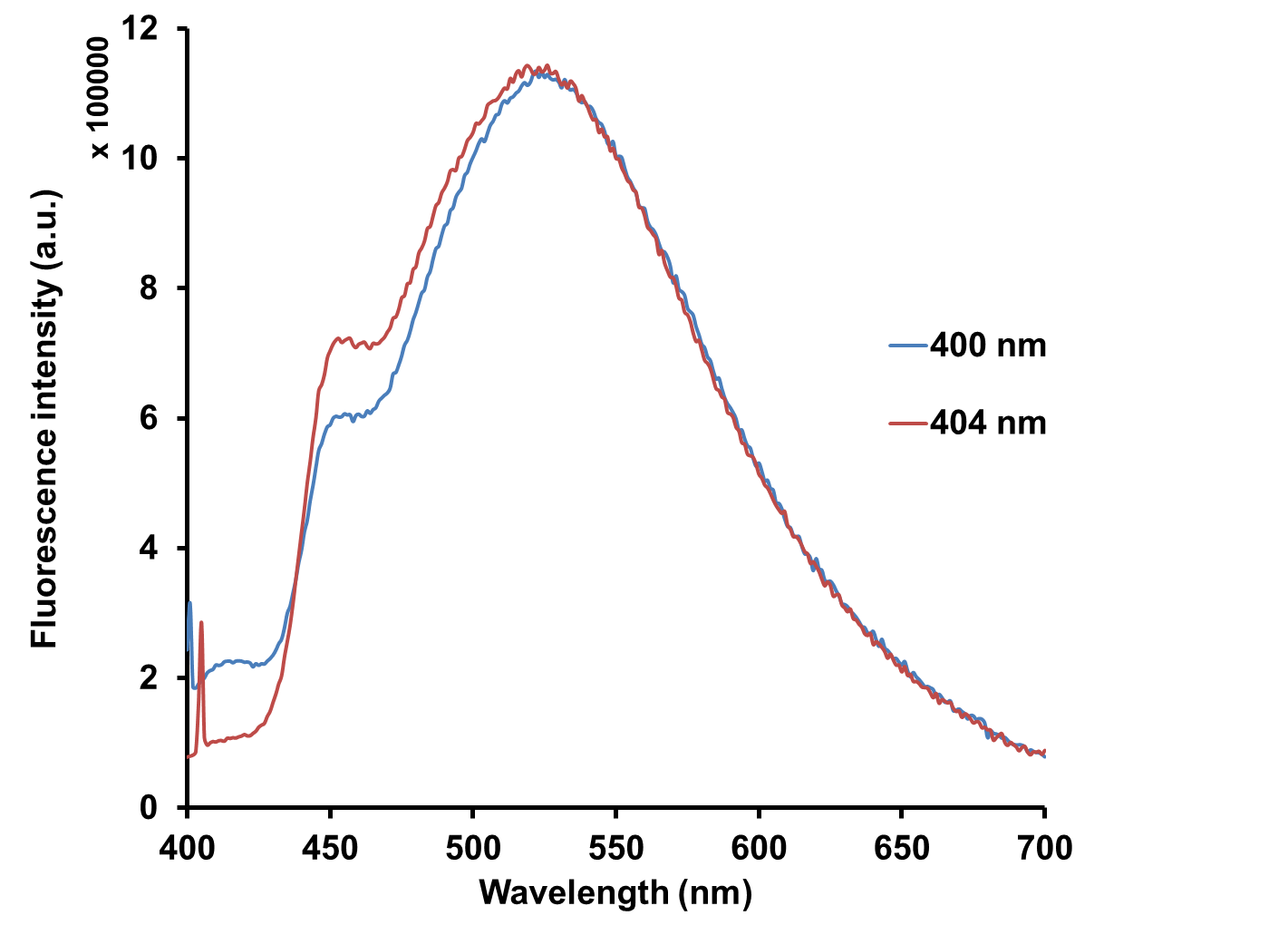
**

**Figure S3.** Fluorescence spectra for ratio mixture (1.375:1:7) at 400 nm and 404 nm.
